# Isolation and Molecular Characterization of *Camelpox* Virus in Dromedary Camels from Outbreak Cases in Borena, Ethiopia

**DOI:** 10.3390/v18060602

**Published:** 2026-05-26

**Authors:** Abdurezak Abrar, Fufa Dawo, Kassaye Adamu, Kenaw Birhanu, Jaleta Shuka, Abinet Legesse, Birhanu Jima, Mirtneh Akalu, Berecha Bayissa, Takele Abayneh

**Affiliations:** 1 Department of Microbiology, Parasitology and Poultry Health, College of Veterinary Medicine and Agriculture, Addis Ababa University, Bishoftu P.O. Box 34, Ethiopia; 2 National Veterinary Institute, Bishoftu P.O. Box 19, Ethiopia; kassayeadamu881@gmail.com (K.A.); kenawbr2129@gmail.com (K.B.); gurmujaletashuka@gmail.com (J.S.); abinetl781@gmail.com (A.L.); birhanujima321@gmail.com (B.J.); mirtneh2010@gmail.com (M.A.); berechabayissa2@gmail.com (B.B.); takeletefera99@gmail.com (T.A.)

**Keywords:** *camelpox*, *Camelus dromedary*, Ethiopia, isolation, molecular characterization

## Abstract

An outbreak investigation of camelpox in dromedary camels (*Camelus dromedarius*) was conducted from October 2024 to May 2025 in Borena Zone, Ethiopia, with the aims of isolating, confirming the etiology and molecular characterization of *camelpox* virus from outbreak cases. The study integrated clinical assessment, virus isolation using Vero cell lines, and molecular characterization using conventional PCR, real-time PCR, sequencing and phylogenetic analysis. Clinically affected camels manifested typical pox-like lesions, fever and swollen lymph nodes, with a morbidity rate of 33.8% (24/71) and a case fatality rate of 4.2% (1/24). The virus was successfully isolated showing typical cytopathic effects of rounding of cells, syncytia, giant cell formation, aggregation and detachment. Conventional PCR targeting A-type inclusion protein (ATIP) gene amplified the expected 881 bp fragment with 26.3% positivity in both skin scab and nasal swab samples. Real-time PCR employing high-resolution melting curve analysis detected the viral DNA in 52.6% of samples with a melting temperature of 73.00 ± 0.20 °C for CMPV. Sequencing of the *ATIP* gene showed 100% nucleotide similarity with reference CMPV strains of CMPV M-96, CMPV CMS, strain 0408151v and CMPV genome (NC_003391), although a single nucleotide variation was noted when compared to the previously reported Ethiopian isolates (KU705085-KU705110) and Israeli isolates (MK910851 and MZ300856), and two nucleotide mismatches were observed with Sudanese isolates (KT931624 and KT931625). Phylogenetic analysis revealed that the current isolates clustered with CMPV strains of CMPV M-96, CMPV CMS, strain 0408151v and others but were distinct from previously reported Ethiopian isolates. This study provides significant insights on early diagnosis and control strategies.

## 1. Introduction

The one-humped camel (*Camelus dromedarius*) is an essential livestock species, with a global population estimated over 42 million, of which 35.6 million are in Africa and 6.76 million in Asia [1]. The one-humped camel accounts for about 95% of the overall Old-World camel population [2]. Africa is home to more than 80% of the world camel population, with the Horn of Africa accounting for 60% of these species. Somalia, Sudan, Ethiopia, Kenya, Djibouti, and Eritrea harbor the highest percentage of one-humped camels in the world [3]. Ethiopia ranks sixth in Africa in terms of camel population, with an estimated 1.75 million camels, predominantly in the arid and semi-arid lowlands of the Somali, Afar, and Southern Oromia regional states [4], where nomadic herders make up the majority of the population [5].

Camels provide meat, milk, wool and transport, playing crucial roles in the livelihoods of pastoral communities. With their special anatomical, physiological and behavioral features that enable them to cope extremely well in the severe environments of aridness, heat and cold. This remarkable adaptability positions them as a key species in addressing the challenges posed by global climate change, offering a sustainable source of nutrition and resources in regions where other livestock may struggle to survive [6].

Despite their resilience, camels are susceptible to camelpox. Camelpox, a highly contagious viral disease affecting camelids, occurs in practically every country where camel husbandry is practiced apart from the introduced dromedary camel in Australia and tylopods (llamas and related species) in South America [7]. The causative agent of camelpox is poxvirus that is taxonomically classified within the genus *Orthopoxvirus* which belongs to the family *Poxviridae*. The family *Poxviridae* is divided into two subfamilies: *Chordopoxviridae*, which infects vertebrates, and *Entomopoxviridae* [8], which are found in insects. Phylogenetic analyses of CMPV show the virus is closely related to the Variola virus, the aetiological agent of smallpox [9]. Outbreaks have been reported throughout the world, including the Middle East (Bahrain, Iran, Iraq, Oman, Saudi Arabia, the United Arab Emirates and Yemen), Asia (Afghanistan and Pakistan), Africa (Algeria, Egypt, Ethiopia, Kenya, Mauritania, Morocco, Niger, Somalia and Sudan) [10], southern parts of Russia and India [11], which are endemic for the disease.

Depending on the virus strain and the animal’s immune status, camelpox is a highly infectious and extremely transmissible disease of skin that affects camels, and it can range from a mild form of skin lesions to severe systemic illnesses [12,13]. The incubation period lasts from nine to thirteen days. The clinical manifestations of camelpox range from mild and inapparent skin-restricted local infections to moderate and severe systemic infections, most likely due to differences in camelpox strains or immunological status of the animals [14]. Fever, enlarged lymph nodes and skin lesions, face oedema, lachrymation, pendulous lips and pox lesions are the disease characteristics [15]. One to three days following the onset of fever, skin lesions appear. They start off as erythematous macules, and then develop to papules and vesicles and ultimately transform into pustules. It is on the ruptured pustules that the crusts form. The head, eyelids, nose and the ear margins are where these lesions initially appear. The entire head may enlarge in severe cases. Skin lesions might later spread to the genitalia, perineum, mammary glands, neck and limbs. In its generalized form pox lesions can spread throughout the body. It might take up to four to six weeks for skin lesions to heal. The systemic form of the disease can cause lesions in the respiratory tract and oral mucous membranes and pox lesions can be observed in these areas [14,16].

Transmission of camelpox occurs via two methods: direct contact with an infected animal or indirect contact with a contaminated environment. Direct transmission occurs when infected animals, with skin lesions, come into contact with vulnerable animals. Additionally, potential vectors like mosquitoes and biting flies can also cause mechanical transfer [12]. Infected camels may shed the virus through scab materials and secretions like milk, saliva, ocular, and nasal discharges into the environment, including water, where they potentially infect vulnerable animals [12,17] and probably human beings. In dry scabs, the virus can survive for up to 4 months. The prevalence of the disease is socioeconomically significant as it causes significant losses in terms of morbidity and mortality, weight loss, abortion and reduced milk production. Outbreaks of camelpox cause substantial economic damage and necessitate quarantine and containment efforts to prevent the disease from spreading [18,19].

Other animal species such as cattle, sheep, and goats are not infected by *camelpox* virus because it shows a higher degree of host specificity toward camels. However, given the small number of human camelpox cases that have been reported, it appears that the disease could be significant for public-health concern [16,20,21]. Bera et al. [20] reported CMPV is a zoonotic disease based on clinical and epidemiological evidence, as well as serological and molecular characterization of the causative agent in three human cases. This was the first incidence of camelpox zoonosis in India as well as in the entire world to be confirmed by laboratory. Khalafalla & Abdelazim [21] provided further evidence of CMPV zoonotic nature through cases in eastern Sudan.

In Ethiopia, camelpox is known to affect camel populations, particularly in arid and semi-arid regions of the country. The disease leads to severe economic losses due to high morbidity and mortality rates, mostly in young camels, as well as reduced productivity in infected herds [22]. Despite the increasing frequency of camelpox outbreak, comprehensive studies focusing on isolation and molecular characterization of CMPV from outbreak cases across different regions and/or locations of Ethiopia remain scarce.

The recurrent nature of camelpox outbreaks in Ethiopia underscores the urgency of identifying the genetic variations in local strains. Although vaccination is available using a live attenuated camelpox vaccine, containing *camelpox* virus strain, its application in Ethiopia is not widely implemented on a routine basis. There are limited documented reports of systematic vaccination coverage in the country. However, vaccination campaigns are conducted by government and non-governmental organizations in response to outbreaks. Understanding the phylogenetic relationships among CMPV isolates is important for the assessment of the current vaccine effectiveness. There are growing concerns that vaccine failure may contribute to continued outbreaks, highlighting the need for in-depth genetic and phylogenetic analyses to support the development of more effective vaccines [23].

Here, we report an outbreak investigation of camelpox in a dromedary herd in the Borana, Yabello districts of Oromia Regional State that occurred in October 2024. The investigation included isolation of the virus and the virus isolates were molecularly characterized by sequencing and compared by phylogenetic analysis.

## 2. Materials and Methods

### 2.1. Study Area

**Figure 1:**
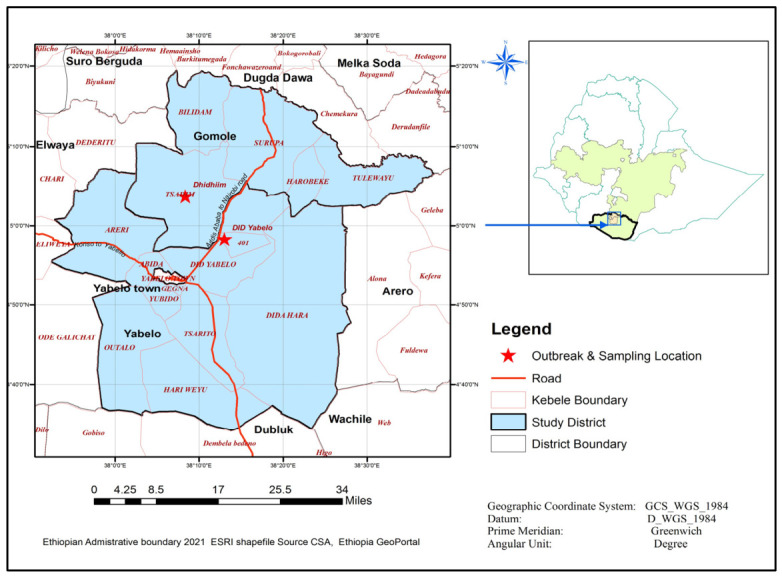
Map showing location of camelpox outbreaks and sampled area.

The study was conducted from October 2024 to May 2025 in selected camel herds in the Borana lowlands of Southern Ethiopia. Borana is located in the lowlands of the Southern part of Oromia, Ethiopia (Figure 1), and is characterized by a semi-arid to arid climate with bimodal rainfall distribution. The long rainy season occurs from March to May, while the short rainy season is from September to November. However, rainfall has become increasingly erratic, leading to frequent droughts and variability in livestock production [24].

The study area is the town of Yabello, where camels made up 4–6% of the total herd on composition handled by the local pastoral communities, which lies at a latitude of 4°53′ N and longitude of 38°5′ E, altitude of 1857 m above sea level and at a distance of 565 km south of Addis Ababa. The area is representative of typical pastoral settings where livestock, particularly cattle, goats, sheep and camels, are raised together [25]. Pastoralists in the area heavily rely on livestock for their livelihood, with about 70% of the population practicing pastoralism. In response to the increasing aridity, many Borana pastoralists have diversified their herds by raising more drought-resilient animals such as camels, goats and sheep [26].

### 2.2. Study Design and Study Population

The purpose of the study was to investigate camelpox outbreaks in camels that occurred in October 2024, in a particular area of Yabello, Borena, namely Dhadiim and Dida Yabello districts. According to outbreaks reports from the district animal health services office, the study population consisted of specifically selected herds with camels. Camels were examined for clinical symptoms of CMPV, such as fever, skin nodules, swollen lymph nodes and lacrimation, and camels manifesting these signs considered positive for CMPV. The age, sex and vaccination history of the animals were also documented. An outbreak investigation approach was used in the study to assess the disease incidence and fatalities in the affected camel herds. Data were gathered through collection techniques observation and interviews with camel owners and experts in animal health. Camels suspected of CMPV were purposively sampled. Descriptive statistics were used to assess the collected data in order to determine the disease’s incidence, mortality and case fatality rates in the affected population.

### 2.3. Clinical Evaluation of Diseased Animals

Clinical data were collected with the help of district animal health workers that are based in Yabello. Camelpox outbreaks were reported in pastoral associations (12) PAs. All affected camels were thoroughly inspected. Samples were taken from skin nodules and nasal swabs and tested for CMPV using routine diagnostic methods such as both conventional polymerase chain reaction (PCR), Real-Time PCR, and virus isolation.

### 2.4. Sample Collection

A total of 19 samples, 5 scabs from skin lesions and 14 swab samples, were collected from camels suspected of camelpox infection in two villages from 2 PAs within Yabello specifically from Dhadiim and Dida Yabello districts, Borena Zone, Oromia Region. Each sampled animal contributed only one sample type; therefore, no paired skin scab and nasal swab samples were collected from the same anima. These villages were selected due to frequent reports of camelpox disease from 12 PAs to the National Veterinary Institute, in herds with a history of camelpox incidents. Skin scalp samples were collected by incising the nodule using a sterile surgical scalpel blade by holding the tissue with forceps. Swab samples were directly collected from the nasal cavity using a sterile swab. All collected samples were transferred to universal tube containing 7.2 pH of phosphate-buffered saline (PBS) with 2% antibiotic (neomycin sulphate and polymyxin B sulphate). The samples were labeled with relevant details, including location, date and clinical signs. All samples were transported on ice in a cool box to the National Veterinary Institute, Bishoftu, and stored at −20 °C until testing or at −70 °C if longer storage was required. All samples were collected in accordance with the procedures approved by the Institutional Animal Care and Use Committee of AAU-CVMA (Approval No. VM/ERC/04/6617/2025) while adhering to established animal welfare guidelines.

### 2.5. Virus Isolation

#### 2.5.1. Sample Preparation

Skin scrapping samples were washed three times in sterile PBS containing antibiotics and an antifungal. An amount of 1 gm of sample was grounded using a sterile pestle and mortar. Tissue homogenates were prepared by adding 10 mL of PBS supplemented with 1% penicillin-streptomycin, and 0.3% Amphotericin B to obtain a 10% (*w*/*v*) suspension. The resulting homogenate was freeze-thawed 2–3 times to enhance the viral release from the cells. The mixture was centrifuged at 1000× *g* for 10 min and the supernatant was carefully collected. To minimize bacterial contamination, the supernatant was filtered through 0.45 μm syringe filter (Millipore, Carrigtwohill, Ireland). The swab samples were clarified by centrifugation at 1000× *g* for 10 min and processed in a similar manner to the skin scraping. All samples were handled following World Organization for Animal Health (WOAH) standard operating procedures [27].

#### 2.5.2. Virus Isolation Using Vero Cells

The supernatant of pathological tissue and swab homogenates were collected, and 1 mL was inoculated onto cells in 25 cm^2^ tissue culture flasks. The cultures were incubated at 37 °C for 1 h to allow viral adsorption, after which the excess of the inoculum was removed. One well was maintained uninoculated as cell control per plate. Subsequently, the cultures were supplemented with maintenance media (Glasgow Minimum Essential Medium with 2% fetal bovine serum (FBS), 1% penicillin-streptomycin, and 0.3% Amphotericin B). The cultures were incubated at 37 °C, 5% CO_2_, and monitored daily for the appearance of cytopathic effects (CPE) for 5–7 days [27]. The samples were frozen upon observing CPE, and samples that did not show CPE after three blind passages were considered negative.

### 2.6. Polymerase Chain Reaction

The pathological tissue and swab homogenates (10% *w*/*v* in PBS) were centrifuged at 1000× *g* for 10 min at +4 °C. DNA was extracted directly from the supernatant of clinical specimens using the DNeasy Blood and Tissue kit (Qiagen) according to the manufacturer’s instructions. Briefly, 200 μL of sample was added to a clean tube, followed by addition of 20 μL of proteinase K and 200 μL of lysis buffer (AL buffer). The mixture was vortexed for 15 s and incubated at 60 °C for 10 min to lyse the cells and release viral DNA. After incubation, 200 μL of 96–100% ethanol was added, and the solution was homogenized. Then, the suspension was transferred to a mini spin column (Qiagen, Hilden, Germnay), and centrifuged at 8000 rpm for 1 min, discarding the flow-through. Next 500 μL washing buffer (AW1) was added to the column and centrifuged at 8000 rpm for 1 min. The flow through was discarded. This was followed by the addition of 500 μL AW2 washing buffer, with subsequent centrifugation at 14,000 rpm for 3 min to thoroughly wash the DNA. The mini spin column was placed in the 2 mL collection tube and centrifuged again for 1 min at 14,000 rpm to dry the column matrix. Finally, DNA was eluted by adding 50 μL elution buffer and centrifuging at 8000 rpm for 1 min. The eluted DNA was labeled and stored at −20 °C until further processing.

PCR assay was performed as described before [27,28] using the primer pair: Fow-5′-AAT-ACA-AGG-AGG-ATC-T-3′ and Rev-5′-CTT-AAC-TTT-TTC-TTT-CTC-3′. These primers amplify an 881 bp fragment within the gene sequence encoding the ATIP region of camelpox, corresponding to the annotated open reading frame CamMLVgp144 in the reference genome (Genbank accession: NC_003391), and are specific for the CMPV. The protocol used allows the detection and differentiation of species of the genus *Orthopoxvirus* because of the size differences in the amplicons.

DNA amplification was carried out using a Qiagen PCR kit that contained a PCR premix microtube. The PCR was carried out in a final volume of 20 μL containing 2 μL of each primer, 3 μL of DNA template and an appropriate volume of nuclease-free water. The samples were incubated in a thermal cycler using the following reaction conditions: 5 min at 95 °C (initial denaturation step), followed by 35 cycles of 1 min at 95 °C, 1 min at 55 °C and 1.5 min at 72 °C, and a final elongation step of 7 min at 72 °C. The temperature was then held at 4 °C until analysis.

The amplicon was visualized by running ten microliters of the PCR products that were mixed with a 6× loading buffer and loaded onto 1.5% agarose gel in TBE (Tris/Borate/EDTA) buffer stained with GelRed. A 100 bp DNA molecular marker ladder was loaded into the first lane for size comparison. The gel was then subjected to electrophoresis at 100 volts for approximately 60 min. Gel documentation using UV transilluminator (UVItec, Cambridge, UK) was used to visualize the DNA bands after electrophoresis, enabling the estimation of PCR product sizes through comparison with the bands of the molecular marker [27].

### 2.7. Differentiation of Orthopoxviruses Using Real-Time PCR

Quantitative PCR (qPCR), based on high-resolution multing curve analysis (HRMCA) using multiplex real-time assays (Table 1), was utilized to differentiate between poxviruses. This method relies on the analysis of PCR amplicons produced with genus-specific primer pairs and double-stranded DNA binding dye, where discrimination is based on the differences in fragment size and GC content. The method generates three well separated melting regions for each genus (*Orthopoxvirus*, *Capripoxvirus*, and *Parapoxvirus*) and the technique also provides additional genotyping of the viruses within each of the three genera; (cowpox virus (CPV) and *camelpox* virus (CMPV) [genus *Orthopoxvirus*]; goatpox virus (GPV), sheeppox virus (SPV) and lumpy skin disease virus (LSDV) [genus *Capripoxvirus*]; orf virus (ORFV), pseudocowpox virus (PCPV) and bovine papular stomatitis virus (BPSV) [genus *Parapoxvirus*]) [29]. The reaction mix was prepared as follows: 2 µL of DNA template, 10 µL of 2× SsoFast™ EvaGreen^®^ Supermix (Bio-Rad), and 0.4 µM each of primer and ddH_2_O, to a final reaction volume of 20 µL. Positive control plasmids representing Orthopoxviruses (CMPV- Hadow/01/2012), and a negative control composed of nuclease-free water were included in each run.

The PCRs and melting curve analysis were performed on the CFX96^TM^ Touch Real-Time PCR Detection System (Bio-Rad Laboratories), following the condition previously described by [29] with a slight modification. Briefly, the initial denaturation step at 95 °C for 4 min was followed by 40 cycles of 1 s at 95 °C, 2 s at 59 °C and 2 s at 72 °C. The PCR products were then denatured at 95 °C for 30 s, cooled at 60 °C for 60 s, and melted from 65 °C to 85 °C with a temperature increment of 0.2 °C every ten seconds and a continuous data recording. The data was analyzed using the CFX Manager ^TM^ Software version 3.0 (Bio-Rad, Hercules, CA, USA) and the Precision Melt Analysis Software version 1.2 (Bio-Rad, Hercules, CA, USA).

### 2.8. Sequencing and Phylogenetic Analysis

The positive PCR products visualized by gel electrophoresis were purified using the Wizard SV Gel and PCR clean-up system kit (Promega, Madison, WI, USA) according to the manufacturer’s instructions and the purified products were quantified spectrophotometrically using the NanoDrop^TM^ 2000c spectrophotometer (Thermo Fisher Scientific, Waltham, MA, USA). The PCR products were sequenced commercially by LGC Genomics (Germany) using Sanger sequencing method. Both the forward and reverse sequencing reaction were conducted using the same primers employed for the PCR amplification.

The obtained nucleotide sequences were inspected for quality and trimmed to remove low-quality bases with Finch Tv (version 1.4.0). The forward and reverse were assembled to generate consensus sequences using BioEdit Software (version 7.2.5). The resulting sequences were compared with the CMPV reference virus sequence (Accession No.: MK910851) using the online NCBI BLAST tool. A representative dataset of Orthopoxvirus sequences including *camelpox* virus, *vaccinia* virus, *horsepox* virus, *rabitpox* virus, *taterapox* virus, and *cowpox* virus, was retrieved from GenBank based on relevance to ATIP locus. Accession numbers of all sequences used are provided in (Table 2). Multiple sequence alignments were performed on BioEdit Software (version 7.2.5) using the ClustalW. The phylogenetic tree was constructed on MEGA Software (version 12.0.11) based on the Neighbor-Joining method and p-distance model was selected as the optimal model for the dataset. The evolutionary history was inferred using the Neighbor-Joining method. The reliability of the inferred phylogenetic tree was tested by performing 1000 bootstrap replicates [30]. The final tree was visualized and edited in FigTree v1.4.

### 2.9. Data Analysis

Clinical findings, disease incidence, mortality and case fatality rates among affected camel herds were summarized using descriptive statistics. Molecular diagnostic results such as PCR and Real-Time PCR outputs were interpreted based on expected amplicon sizes. Bioinformatics tools were used to align and compare sequenced data, and the phylogenetic analysis was determined through the construction of a Neighbor-Joining method tree using MEGA Software (version 12.0.11). This analysis provided the genetic similarity between local isolates and reference CMPV strains from GenBank.

### 2.10. Ethical Statement

Ethical approval for the study was obtained from the Institutional Review Board of Addis Ababa University College of Veterinary Medicine and Agriculture (Reference No.: VM/ERC/04/6617/2025, approval date: 25 February 2025). Throughout the study, all the methodologies were aligned with this ethical and regulatory framework to ensure the highest levels of animal welfare and ethical conduct.

## 3. Results

### 3.1. Clinical Observations

**Figure 2:**
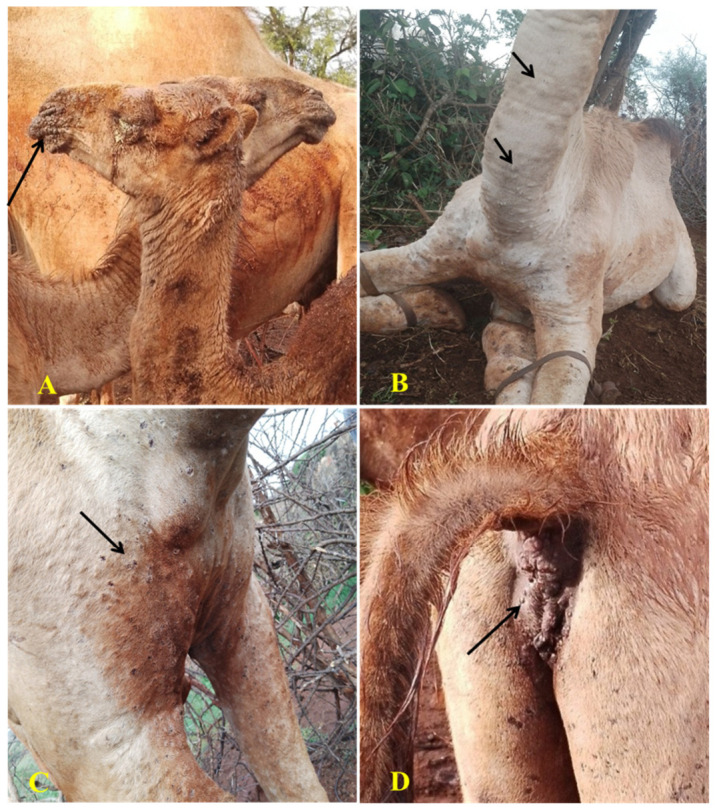
Clinical observations of camelpox-specific lesions. (**A**): Skin lesions on the mouth of young camel, (**B**): unruptured skin nodules on the neck and leg areas, (**C**): adult camel showing ruptured pustules throughout the body, (**D**): skin lesion on the genital and perineal areas.

Clinical investigations of the one-humped camels (*Camelus dromedarius*) reared in the two districts found in Yabello, Borena Zone, Ethiopia, showed pox-like skin lesions (Figure 2). According to field reports submitted to the National Veterinary Institute in October 2024, 46 diseased camels and 13 deaths were recorded across the outbreak areas. Affected camels exhibited typical signs of camelpox, such as fever and skin lesions on the head, eyelids, nostrils and the margins of the ears, neck, limbs, genitalia and perineum and inguinal regions. The disease was observed in all age and sex groups with similar skin lesions. Although lesions were observed in both age groups, young camels showed more severe clinical signs, while adult camels were more frequently affected (Figure 2). Among the 19 clinically examined camels, 13 (68.4%) were females and 6 (31.6%) were males. Based on age classification, 7 (36.8%) were young camels (<5 years), while 12 (63.2%) were old camels (≥5 years). Most of the camels had high fever (39–40 °C) and nodular skin lesions. Cases of abortion were also reported in some PAs. The morbidity rate was 33.8% in the visited outbreak areas. Among 71 camels in Yabello, 24 (33.8%) cases were clinically sick of camelpox, with 2 severe cases and 21 recoveries (91.7%). Only one fatality (CFR = 4.2%) was recorded, despite all herds being vaccinated. In general, the percentage of infected camels varied between herds, ranging from 11.5 to 60; detailed data of the affected herds is summarized in (Table 3).

### 3.2. Isolation of Virus in African Green Monkey Cells (Vero Cell Lines)

**Figure 3:**
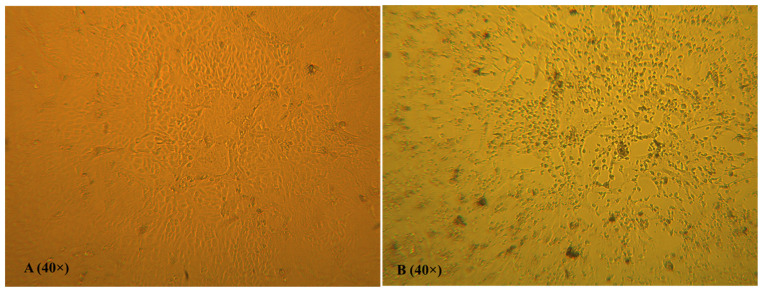
Virus isolation on Vero cells (40× magnification). Uninfected cells maintained normal cell morphology (**A**), while inoculated cells showed morphological changes suggestive of cytopathic effect four days after inoculation (**B**).

All of PCR confirmed CMPV-positive samples were inoculated on Vero cell cultures. The isolated CMPV produced the cytopathic effect (CPE) after the second passages from 4 to 7 days post inoculation. The CPE was characterized by cell rounding, syncytia, giant cell formation, aggregation and detachment of the cell sheet; nonetheless, the negative control cell remained unchanged, as shown in (Figure 3).

### 3.3. Conventional PCR

**Figure 4:**
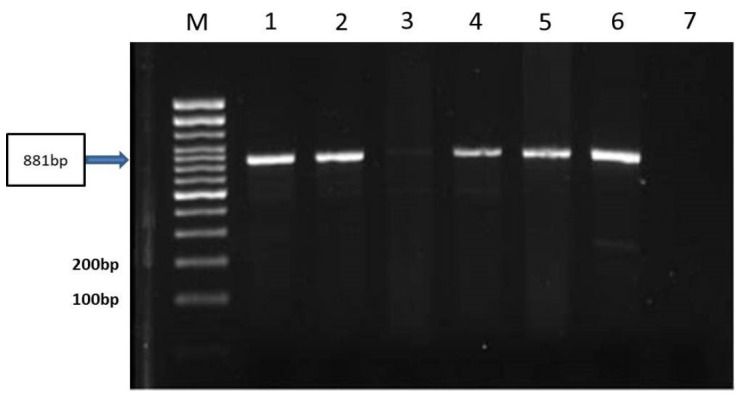
Results of PCR products of *ATIP* gene: Lane M: DNA molecular marker, Lanes 1–5 are skin nodules and swab samples from field, Lane 6: Positive control and Lane 7: Negative control.

Conventional PCR testing of skin scab and nasal swab from camels suspected of CMPV disease yielded amplification products of the expected size (881 bp) corresponding to partial fragments of *ATIP* gene (Figure 4). Of the 19 representative clinical samples selected from a total of 71 suspected camelpox cases across two districts of Yabello, all 5 skin scab samples (100%; 5/5), representing 26.3% of the total samples, and 5 of the 14 nasal swabs (35.7%; 5/14), also representing 26.3% of the total samples, tested positive for *camelpox* virus genome. The 881 bp PCR fragments were successfully amplified in 10 CMPV-positive samples.

### 3.4. Real-Time PCR

**Figure 5:**
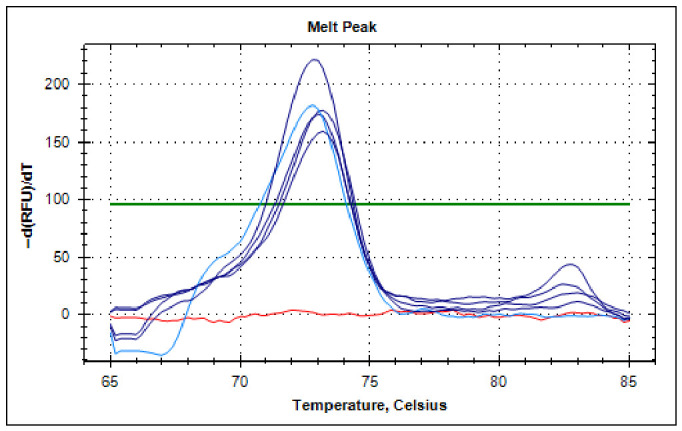
HRM detection of *camelpox* virus diseases. Skin and nasal swab samples of camels tested by HRM assay. Light blue indicates positive control for CMPV displaying a unique melting peak; four samples from Yabello, clustering with the control, are shown in dark blue. The green horizontal indicates the fluorescence threshold used for melt peak detection, while the red line represents the negative control/background signal, confirming the absence of specific amplification.

We detected CMPV DNA in ten out of nineteen samples, with (52.6%) of the samples tested positives using the HRM Assay, of which five were tissue and five were swab samples. The melting temperature of the samples was recorded: CMPV *Tm* (73.00 ± 0.20 °C). The amplification curves corresponding to CMPV-positive samples from Borena, Yabello are shown in (Figure 5); there was no amplification corresponding to *Capripoxvirus* and *Parapoxvirus*.

### 3.5. Molecular Characterization and Phylogenetic Analysis

Of the ten positive samples, only five samples yielded a sufficient amount of DNA for further sequencing. The sequences were analyzed and deposited in the GenBank with the following (GenBank accession numbers: PV737715-PV737719). These isolates revealed no nucleotide variation among themselves, showing that they are highly identical. The partial sequence of this gene showed 100% nucleotide sequence similarity with the reference sequences of CMPV strains found in Mangystau oblast in Kazakhstan (GenBank accession number: AF438165), Iran (GenBank accession number: AY009089), CMPV strain 0408151v (GenBank accession number: KP768318) and CMPV genome (GenBank accession number: NC_003391). The pairwise nucleotide identity analysis is presented in Supplementary Figure S1.

**Figure 6:**
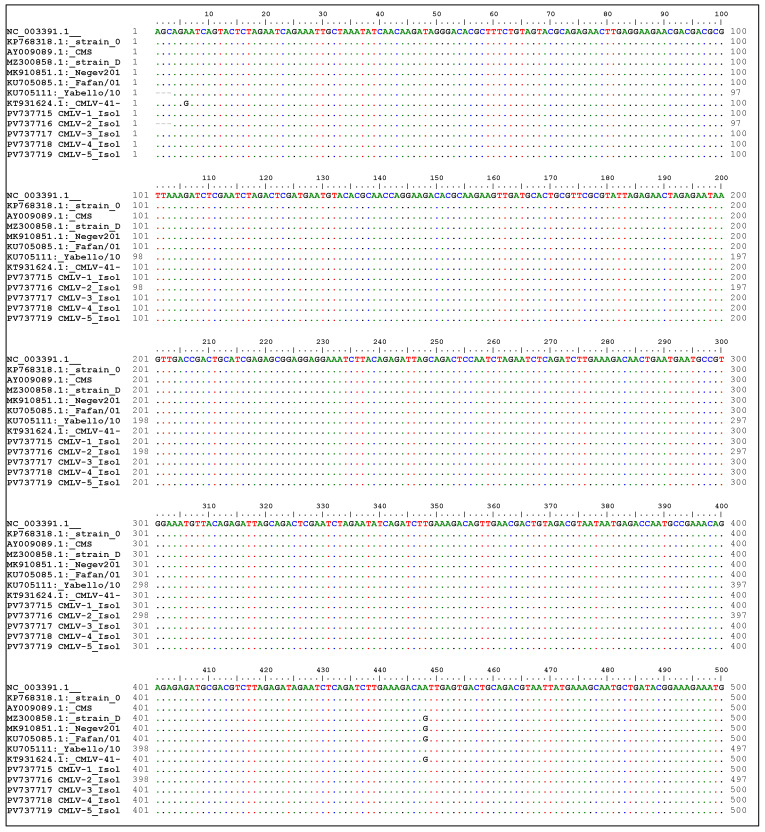

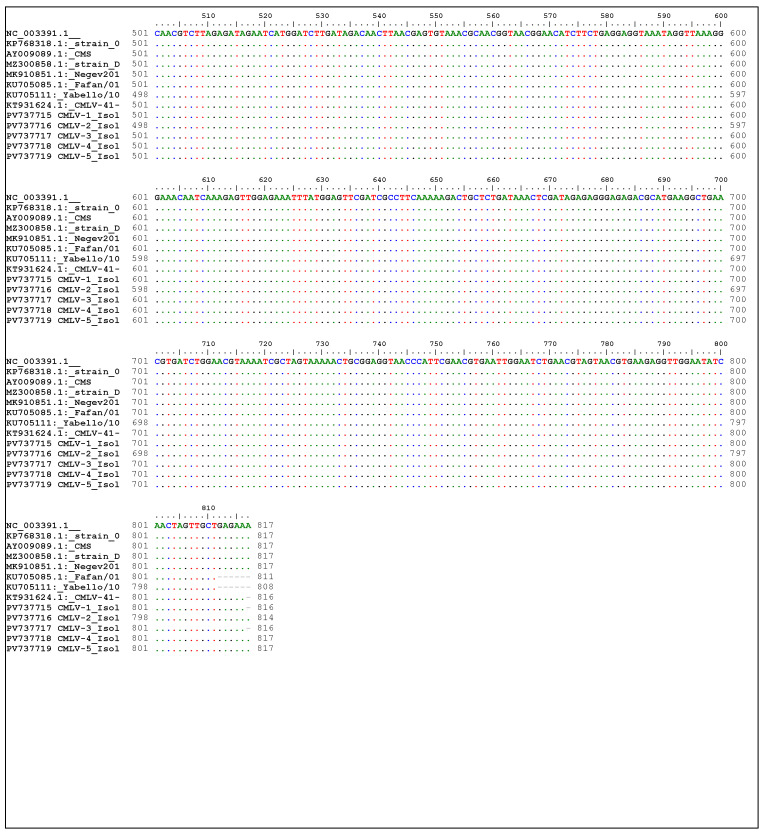
Multiple sequence alignment of CMPV isolates from Borena and homologous CMPV sequences retrieved from GenBank, using *ATIP* gene sequences.

Multiple nucleotide sequence alignment of the partial *ATIP* gene sequences of these five Ethiopian CMPV isolates, alongside homologous CMPV sequences retrieved from GenBank (Figure 6) revealed a single nucleotide variation at position 448. Specifically (A:G) variation was observed at position 448 when compared to CMPV isolates from Israel (MK910851 and MZ300856), and previously reported Ethiopian CMPV isolates (KU705085-KU705110). Additionally, alignment with Sudanese isolates (KT931624 and KT931625) revealed two nucleotide mismatches A:G at nucleotide 6 and 448.

At amino acid level, all five isolates showed 100% identity with A-type inclusion protein partial genes of CMPV (GenBank accession numbers: AOC59220, AMR98480, AXO77476, AMR98476, AMR98478, QCW07459, NP_570534, Q05482) while minimum identity of 93.46% with MPXV gp137 protein of monkey poxvirus (GenBank accession number: WNN25674).

**Figure 7:**
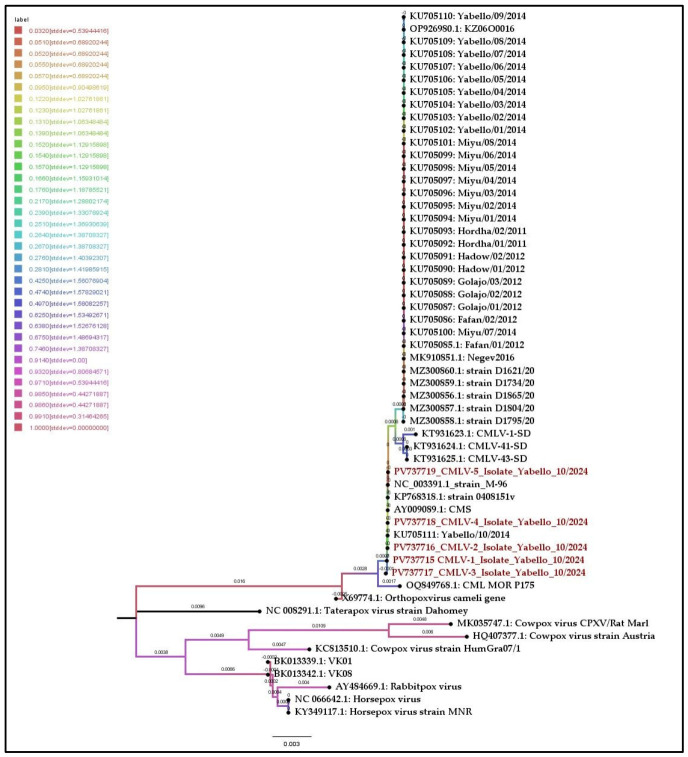
Phylogenetic analysis of camelpox isolates from Borena using *ATIP* gene sequences from various poxviruses retrieved from GenBank.

A total of 56 *ATIP* gene sequences submitted to GenBank were used for phylogenetic analysis of CMPV isolated from Borena (Figure 7). The phylogenetic analysis revealed that all five isolates from Yabello, Borena clustered with CMPV strains 0408151v, M-96 and CMS rather than to other Orthopoxviruses with high bootstrap value.

## 4. Discussion

The Borana community includes both pastoralists and agro-pastoralists, which primarily rely on a combination of livestock rearing and crop cultivation to sustain their livelihood. The region experiences frequent cross-border interactions with neighboring Kenya and Somalia through grazing, livestock trade and smuggling of animal and human drugs. Animal husbandry in the region follows an extensive system, with seasonal mobility and herd management strategies that may involve moving herds to better forage or water points, or splitting herds by keeping lactating and young animals near homesteads while the rest move to distant areas for grazing [24].

*Camelpox* virus in camels is caused by a smallpox-like illness known as camelpox disease. With the exception of the introduced dromedary camel in Australia and tylopods, the disease is enzootic in almost all regions where camel husbandry is practiced (llamas and related species) in South America [7], and is responsible for severe economic losses. Although it is genetically the closest known virus to variola virus, the etiologic agent of smallpox, CMPV remains poorly studied [31]. In the present study *camelpox* virus was successfully cultured and isolated molecularly detected and the *ATIP* gene was sequenced and the evolutionary relationship with other poxviruses was determined using samples from outbreak cases in affected pastoral associations in Yabello, Borena.

The present outbreak occurred in mid-October which is a short rainy month in the Borena Zone of Oromia. According to several studies, the incidence of more severe forms of camelpox outbreaks increases during the rainy season [22,32]. Adult camels were primarily affected by the disease, which manifested skin lesions, typically on hairless areas such as the face, neck, genitalia, mammary glands and perineum, while in several cases, the lesions spread throughout the body. In the current study, the typical clinical symptoms of camelpox such as fever, anorexia, swollen lymph nodes, weakness, lack of appetite, abortion, mortality lacrimation and pustular skin lesions were observed which coincided with several reports by other investigators [7,12,33,34,35,36].

The morbidity and mortality rate for the entire population were 33.8% and 1.4% respectively, but 4.17% (*n* = 1 out of 24) of the clinically affected camels died of generalized camelpox. This outbreak investigation result differs from that of Joseph et al. [35], who reported morbidity and mortality rates of 1.10% and 0.10%, respectively, in a large population of over 5000 dromedary camels in the United Arab Emirates. The difference could arise from variation in sample size, herd management practice, population immunity or environmental conditions. While the UAE outbreak affected a highly controlled commercial herd with a history of sporadic occurrence, the present study involved small camel herds in a pastoral system, where variability in immune response and poor pastoral management could have induced the disease. Additionally stress factors, parasite burdens and the timing of the outbreak within the rainy season could have contributed to increased severity and spread of the disease among the affected herds.

The present study’s results interestingly showed that our primary attempt to isolate tissue specimens from the suspected camelpox lesions in Vero cells was successful, producing a typical cytopathic effect. The clear CPE formation in the Vero cells in the present study confirmed the report by Mosadeghhesari et al. [7], who reported that virus from scab samples collected from clinically sick camels grows well in Vero cells. They demonstrated that the CPE produced by CMPV resulted in rounding of the cells, plaque formation, cytoplasmic elongation and multinucleated giant cell formation in all cell cultures.

The confirmatory diagnosis of the suspected *camelpox* virus was made by conventional PCR performed according to the method described by Meyer et al. [36] where specific 881 bp amplification products were obtained. These findings agree with some research reports [7,34,37,38] in using PCR ATIP position 881 bp which were identified for all of their isolates. In contrast, Yousif & Al-Naeem [38] reported the recovery of about 1500 bp from camelpox vaccine strain at the same position, attributing the difference to sequence variation or insertion events within the target region, possibly linked to vaccine attenuation. Similarly, Ayelet et al. [22] reported comparable variations, reporting that five of their Afar region samples produced a shorter sequence (about 486 bp) length, amplified only by reverse primer; this shorter fragment might be due to occurrence of gene deletion or change at the point of forward primer attachment site.

Our findings showed the relevance of molecular methods for differential diagnosis and the management of pox diseases in camels. This robust HRM assay enabled not only the detection and differentiation *camelpox* virus from other *Orthopoxvirus*, but also other poxviruses, i.e., GTPV, SPPV, and LSDV in the *Capripoxvirus* genus, and ORFV, PCPV, and BPSV in the *Parapoxvirus* genus in a single test that would not have been discovered otherwise.

Multiple sequence alignment of the partial *ATIP* gene showed 100% nucleotide similarity between the present Ethiopian *camelpox* virus isolates and reference strains CMS, M-96, strain 0408151v, isolate of Kazakhistan (NC_003391), and KU705111 which is a local isolate reported by [39]. This finding suggests that there is a close genetic relationship between the Ethiopian CMPV isolates and those from Central Asia and the Middle East, further implying a common ancestral origin among geographically distant strains. However, nucleotide variations were observed in specific positions. A single substitution at position 448: an (A:G) was detected when compared with previously reported Ethiopian isolates (KU705085-KU705110), and Israeli isolates (MK910851 and MZ300856). Furthermore, comparing with Sudanese isolates (KT931624 and KT931625) revealed two nucleotide mismatches at position 6 (A:G) and 448 (A:G).

A phylogenetic tree was constructed to determine the genetic relationships between the current Ethiopian field isolates identified in this study and other isolates retrieved from GenBank. The phylogenetic analysis revealed that the current Ethiopian isolates are distinct from previously reported Ethiopian strains described by [39]. Therefore, this highlights the presence of genetically related but evolutionarily unique local viral strains within Ethiopia. However, the main weakness of this phylogenetic analysis was based on a partial sequence of a single gene (*ATIP*). Such an approach may not fully capture the overall genomic diversity or evolutionary history of the virus. Compared to whole genome analysis, partial gene sequences provide limited phylogenetic resolution and may reduce the accuracy of inferred relationship. Despite these limitations, the molecular characterization presented in this study contributes valuable insights into the genetic diversity of CMPV in Ethiopia. These findings provide useful information for designing a CMPV vaccine that can match strains of the virus that are circulating in Ethiopia.

## 5. Conclusions

The present study confirmed an outbreak of camelpox among dromedary camels in Borena Zone, Ethiopia, through clinical evaluation, virus isolation using Vero cells, conventional PCR, Real-Time PCR, and molecular characterization of the *ATIP* gene. According to the genetic analysis of the study there is a high similarity between CMPV strains circulating in the study and those that have been previously reported in Kazakhstan and Iran, indicating a close phylogenetic relationship and possible trans-boundary transmission patterns.

This research highlights camelpox as one of the most significant viral diseases affecting camel herds in the pastoral regions of Ethiopia. This study not only provided the genetic evidence supporting the classification of Ethiopian CMPV isolates but also it has contributed valuable insights into their molecular characteristics. The molecular methods employed proved to be rapid and reliable diagnostic tool, helping accurate detections, characterization and differentiation of CMPV from other Orthopoxviruses, Capripoxviruses and Parapoxviruses.

The systemic form of the disease observed in the camels and the associated mortality underscore the importance of early diagnosis and intervention. The nomadic lifestyle of camel herd keepers coupled with limited diagnostic facilities and irregular disease reporting, contributes to the silent spread and persistence of the virus in endemic regions. The economic impact of camelpox is enormous, which can lead to production losses, mortality, abortions and reduced milk yield.

Although CMPV primarily infects camels, it is closely related to variola virus, the causative agent of smallpox. Although rare, human infections of CMPV have been previously reported. However human infections of CMPV have not been studied in Ethiopian pastoral communities, emphasizing the need for increased attention, strengthened surveillance and improved diagnostic capacity. Future studies should focus on whole-genome analysis of CMPV strains from different clinical forms to better understand virulence and support vaccine development and control strategies in Ethiopia.

This manuscript is derived from the first author’s M.Sc. Thesis at Addis Ababa University [40].

## Figures and Tables

**Table 1:** Primers used for the HRM assay.

Method	Primer Name	Primer Sequence	Amplicon Size	Target and References
HRM	CaPV-HRM-For	TCCTGGCATTTTAAGTAATGGT	100	Capripoxviruses [29]
	CaPV-HRM-Rev	GTCAGATATAAACCCGGCAAGTG		
HRM	PPV-HRM-For	TCGAAGATCTTGTCCAGGAAG	112	Parapoxviruses [29]
	PPV-HRM-Rev	CCGAGAAGATCAACGAGGTC		
HRM	OPV-HRM-For	AGGACTAGCCGCGGTAACTTT	56	Orthopoxviruses [29]
	OPV-HRM-Rev	ACAAGATAGAAGCGATGGATACTT		

**Table 2:** List of Orthopoxvirus sequences retrieved from GenBank with their accession number.

Organism	Isolate Name	Accession No.
*Camelpox* virus	M-96	NC_003391.1
*Camelpox* virus	strain 0408151v	KP768318.1
*Camelpox* virus	CMS	AY009089.1
*Camelpox* virus	Negev2016	MK910851.1
*Camelpox* virus	D1795/20	MZ300858.1
*Camelpox* virus	D1621/20	MZ300860.1
*Camelpox* virus	D1734/20	MZ300859.1
*Camelpox* virus	D1865/20	MZ300856.1
*Camelpox* virus	D1804/20	MZ300857.1
*Camelpox* virus	CMLV-1-SD	KT931623.1
*Camelpox* virus	CMLV-41-SD	KT931624.1
*Camelpox* virus	CMLV-43-SD	KT931625.1
*Camelpox* virus	CML MOR P175	OQ849768.1
*Camelpox* virus		KU705085.1-KU705111
Orthopoxvirus cameli		X69774.1
*Camelpox* virus	KZ06O0016	OP926980.1
*Vaccinia* virus	VK01	BK013339.1
*Camelpox* virus	CMLV-1, Yabello/ETH/10-2024	PV737715
*Camelpox* virus	CMLV-2, Yabello/ETH/10-2024	PV737716
*Camelpox* virus	CMLV-3, Yabello/ETH/10-2024	PV737717
*Camelpox* virus	CMLV-4, Yabello/ETH/10-2024	PV737718
*Camelpox* virus	CMLV-5, Yabello/ETH/10-2024	PV737719
*Vaccinia* virus	VK08	BK013342.1
*Horsepox* virus		NC_066642.1
*Horsepox* virus	strain_MNR	KY349117.1
*Rabbitpox* virus		AY484669.1
*Cowpox* virus	strain_HumGra07/1	KC813510.1
*Taterapox* virus	strain_Dahomey	NC_008291.1
*Cowpox* virus	CPXV/Rat Marl	MK035747.1
*Cowpox* virus	strain Austria 1999	HQ407377.1

**Table 3:** Information on the samples from which specimens were collected for this study.

Owners Name	No of Animals	No of Diseased	% of Cases	Severe Cases	Recovered Cases	No of Deaths	CFR%	No of Samples	Vaccination History
Herd 1	19	7	36.8	1	6	0	0.0	5	Vaccinated
Herd 2	15	9	60.0	0	8	0	0.0	8	Vaccinated
Herd 3	11	5	45.5	1	4	1	20	3	Vaccinated
Herd 4	26	3	11.5	0	3	0	0.0	3	Vaccinated
Total	71	24	33.8	2	21	1	4.17	19	-

## Data Availability

The data presented in this study are included in the manuscript or are available on request from the corresponding authors. The isolated virus genes sequenced in this study have been deposited in GenBank using the accession numbers: PV737715-PV737719.

## Supplementary Materials

The following is available online at https://doi.org/10.5281/zenodo.19338964, accessed on 20 March 2026.
